# Impact of COVID-19 Pandemic on Trauma CT Imaging

**DOI:** 10.1155/2022/9596148

**Published:** 2022-06-02

**Authors:** Yi Yan, Kai Hu, Kevin Shek, Jun Li, Shady Attalla, John Ross Bonanni, Jai Jai Shankar, Lisa McPhee

**Affiliations:** ^1^Rady Faculty of Health Sciences, Radiology Department, University of Manitoba, GA216-820 Sherbrook Street, Winnipeg R3A 1R9, MB, Canada; ^2^Radiology Department, Radiology and Diagnostic Imaging, St. Boniface General Hospital, 409 Taché Ave, Winnipeg R2H2A6, MB, Canada; ^3^Department of Neurology, Xiangya Hospital, Central South University, Xiangya Road 87, Changsha 410008, Hunan, China; ^4^Department of Biological Sciences, Brock University, 1812 Sir Issac Brock Way, St.Catharines, Ontario L2S 3A1, Canada; ^5^Ain Shams University, Ramsis Street, Abbassia Square, Cairo 11566, Egypt; ^6^Pediatric Radiology, Max Rady College of Medicine Radiology, University of Manitoba, GA216-820 Sherbrook Street, Winnipeg R3T 2N2, MB, Canada

## Abstract

**Purpose:**

The goal of this study was to understand the impact of COVID-19 pandemic and associated lockdown measures on the volume, rate, and type of trauma presenting to the emergency department (ED) by using trauma-initiated CT studies to capture patient data.

**Materials and Methods:**

We performed a retrospective observational study comparing patients undergoing CT scans for trauma during the 1^st^ and 2^nd^ lockdown periods compared to corresponding prepandemic months. During two lockdown periods, public places such as restaurants, libraries, parks, and shops across the province were shut down. Government-led messaging advised that people should stay at home and practice social distancing. The rate of trauma-initiated CT scans and the proportion of different types of traumas were compared between time periods.

**Results:**

There was no significant difference in overall trauma-initiated CT scans between the prepandemic and pandemic levels. Motor vehicle collision (MVC) cases decreased from 18.2% to 15.6% during the first lockdown period (*p* = 0.049) and also reduced from 29.1% to 25.2% during the second lockdown period (*p* = 0.013). Trauma from falls increased from 19.1% to 27.5% (*p* = 0.036) during the first lockdown, despite no significant change during the 2^nd^ lockdown. Furthermore, the percentage of stab injuries increased from 25.0% to 38.9% while blunt trauma decreased from 68.5% to 54.3% during two lockdowns (*p* = 0.015).

**Conclusion:**

The total number of trauma-initiated CT scans did not significantly decrease during the lockdown periods. Stabbings and falls increased during lockdown periods while MVCs and blunt trauma decreased.

## 1. Introduction

The coronavirus disease 2019 (COVID-19) pandemic was caused by a coronavirus called SARS-CoV-2. The COVID-19 pandemic has challenged the capacity and capability of health care systems globally [1]. Hospitals have been reorganized and repurposed to isolate and care for SARS-CoV-2 positive patients [2]. However, medical and surgical emergencies, including trauma, continually occur in our communities [2, 3]. Both the European Society of Trauma and Emergency Surgery (ESTES) and the American College of Surgeons (ACS) have warned of the possibility of the pandemic impacting the care of trauma patients. While overall ED visits have declined during the pandemic [4], there have been relatively few studies assessing the change in volume and type of trauma patients presenting to the ED during lockdown time periods [5–8]. A better understanding of trauma patterns during the pandemic and lockdown periods will help inform healthcare policy and resource allocation.

We aim to better understand the volume, rate, and types of trauma presenting to our level 1 Provincial trauma center by comparing trauma-initiated CT studies during COVID-19 pandemic lockdown time periods to a pre-COVID-19 pandemic period.

## 2. Materials and Methods

### 2.1. Study Design

**Figure 1:**
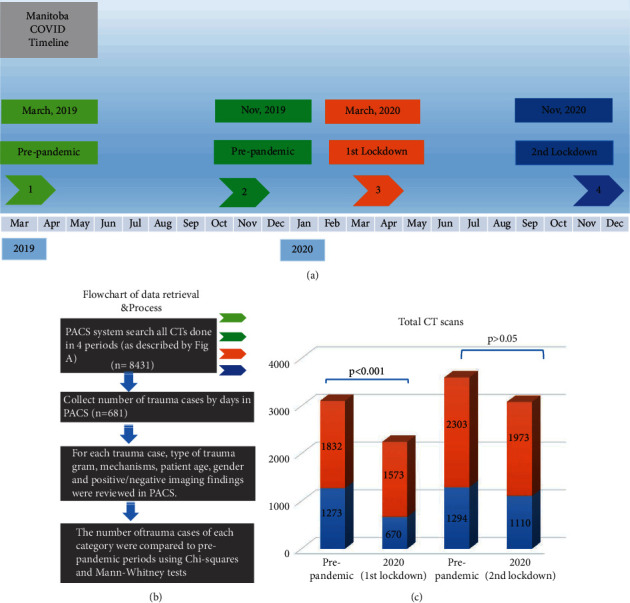
(a) Timeline of the COVID-19 Pandemic in Manitoba. Period 1: a prepandemic month in 2019 (March); Period 2: a prepandemic month in 2019 (Nov); Period 3: a 1-month period during the 1st COVID-19 lockdown period in 2020(March); Period 4: A 1-month period during the 2nd lockdown in 2020 (Nov) (Figure 1(a)). We matched the same month during prepandemic control due to a known seasonal variability on CT volume (winter road conditions, ice, and snow). (b) The flowchart depicting the process of a total of 962 trauma patients selected in our study. The demographics and subtypes of scans were collected via the PACS system. (c) The comparison of total elective CT scans and STAT CT cases including emergency and inpatient cases in our level 1 trauma center during two COVID-19 lockdown periods in 2020 and corresponding prepandemic periods in 2019, was made using *χ*2 test (Chi-square analysis). *p* value refers to a relative portion of STAT cases to total CT cases.

A retrospective observational study was performed. Data were collected at the level 1 provincial trauma center in Winnipeg. Government-led messaging has been delivered that people should stay at home or practice social distancing if they go outside for exercise, shop for essentials, or look after other vulnerable people. Places such as restaurants, libraries, parks, and shops across the province were shut down beginning on March 12, 2020, and ending on May 5, 2020. The 2^nd^ lockdown started on November 2 and ended on December 12, 2020, with similar restrictions. Our data were collected over four time periods including both locking down periods and corresponding prepandemic months: Period 1: a prepandemic month in 2019 (March); Period 2: a prepandemic month in 2019 (Nov); Period 3: a 1-month period during the 1^st^ COVID-19 lockdown period in 2020 (March 12-April 12); Period 4: a 1-month period during the 2^nd^ lockdown in 2020 (Nov 2^nd^ to Dec 2^nd^) (Figure 1(a)). We matched the same month during prepandemic control due to a known seasonal variability on CT volume (winter road conditions, ice, and snow).

The number of elective CT and STAT CT (including ED and inpatient scans) was collected via the institutions' PACS system (picture archiving and communication system) using simple searching criteria (individual date in these periods and modality: CT). The number of STAT CT cases ordered from the ED and hospital wards was calculated in PACS. Each CT requisition was reviewed. The cases specifically ordered for trauma presenting to the emergency department such as, MVC, falls, and assaults were considered as trauma CT cases and included in our studies. The number of total CT scans ordered for trauma and specific types of CTs was calculated using the breakdown described below.

### 2.2. Data Collection and Extraction

After reviewing consecutive radiology reports and requisitions, the following information was collected from PACS: patient age, type of scan, mechanism of injury, and injuries on CT (Figure 1(b)). Five subcategories of trauma are defined as follows:1) blunt trauma; (2) stabbing (all stabbings with selfinflicted have been taken into consideration separately); (3) gunshot wound (GSW); (4) fall; (5) motor vehicle collision (MVC) involving drivers, passengers, and pedestrians; and (6) others (any off-road vehicles such as ATV and/or snowmobiles) were documented separately.

As per institutional protocol, six different types of CT scans were ordered by ER physicians for trauma patients and subsequently performed in the CT department: (1) pan-scan (including noncontrast CT brain and cervical spine, CT angiogram chest, CT portal venous phase of abdomen/pelvis); (2) separate head CT including head or/and facial bone or/and cervical spine; (3) separate CT angiogram chest; (4) separate CT abdomen with venous phase; (5) CT angiogram run-off of lower and/or upper extremities or angiogram of neck/carotid; and (6) MSK studies including any bony or soft tissue injuries.

A trauma CT scan was considered positive when there was any acute organ, osseous, vascular, or soft tissue injury. Findings that were incidental and of doubtful clinical significance were not considered as positive scans. Any injuries related to bone insufficiency fractures were excluded. All age groups were included.

### 2.3. Statistical Analysis

The percentage of each category between the two-month lockdown periods during the COVID-19 pandemic in 2020 and the prepandemic month in 2019 was compared using the Mann–Whitney *U* test for nonnormally distributed continuous variables and *χ*2 test (chi-square) for categorical variables. *p* value and 95% confidence intervals (CI) were calculated. The strength of exposure factors to trauma was also calculated using odds ratio. All analyses were performed using SAS 9.4 (SAS Inc, Cary, NC, USA).

## 3. Results

### 3.1. Total Urgent and Elective CT Scans during the COVID-19 Pandemic

A comparison of the number of CT scans in our level 1 trauma center was made between the first and second lockdown months and corresponding prepandemic months (Period 1 versus 3, and Period 2 versus 4). During the first lockdown month, the total number of scans performed (including STAT and elective cases) decreased by 41% from 3105 to 2243. The STAT (urgent) cases including emergency and inpatient cases decreased by 18.8% from 1832 to 1573 (Figure 1(c)). The relative portion of STAT cases increased from 59.0% to 70.1% (*p* < 0.001, chi-square test) (Figure 1(c)). During the 2^nd^ lockdown period (Period 3), the relative portion of STAT cases was not significantly changed as compared to the prepandemic month (64% versus 64%, *p* > 0.05, chi-square test, Table 1 and Figure 1(c)).

### 3.2. Trauma Cases and Trends during the COVID-19 Pandemic

**Figure 2:**
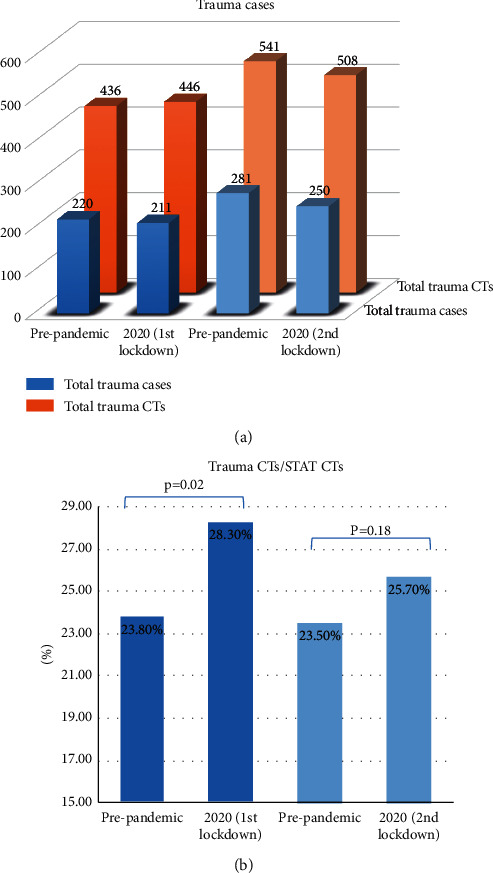
(a) The total number of trauma cases and trauma-related CT scans in our level 1 trauma center in two COVID-19 lockdown periods in 2020 versus the prepandemic period in 2019. (b) The comparison of the relative ratio of trauma CT scans to ED CT scans among two lockdown periods as compared to control prepandemic months.

There was no significant difference of total trauma cases (220 vs 211 and 281 vs 250) (*p* = 0.32) and trauma-related CT scans (436 vs 446 and 541 vs 450) (*p* = 0.56) among two lockdown periods as compared to corresponding prepandemic control months (Figure 2(a)). However, the relative percentage of trauma cases presenting to the ED increased significantly from 23.8% to 28.4% during the 1st lockdown period (*p* = 0.02) and also rose from 23.5% to 25.7% during the 2^nd^ lockdown despite no statistical significance (*p* = 0.18) (Figure 2(b)). There was an upward trend in the rate of pan-scans performed for trauma during the first lockdown period from 31.8% to 38.4%, but this was not statistically significant (*p* > 0.05) (Table 1). There was a nonsignificant decrease in the percentage of negative CT scans during the first and second lockdown periods from 45.9% prepandemic to 39.8% and then from 52.7% prepandemic to 37.2% (*p* = 0.27) (Table 1).

### 3.3. Characteristics of Trauma Breakdown Categories

**Figure 3:**
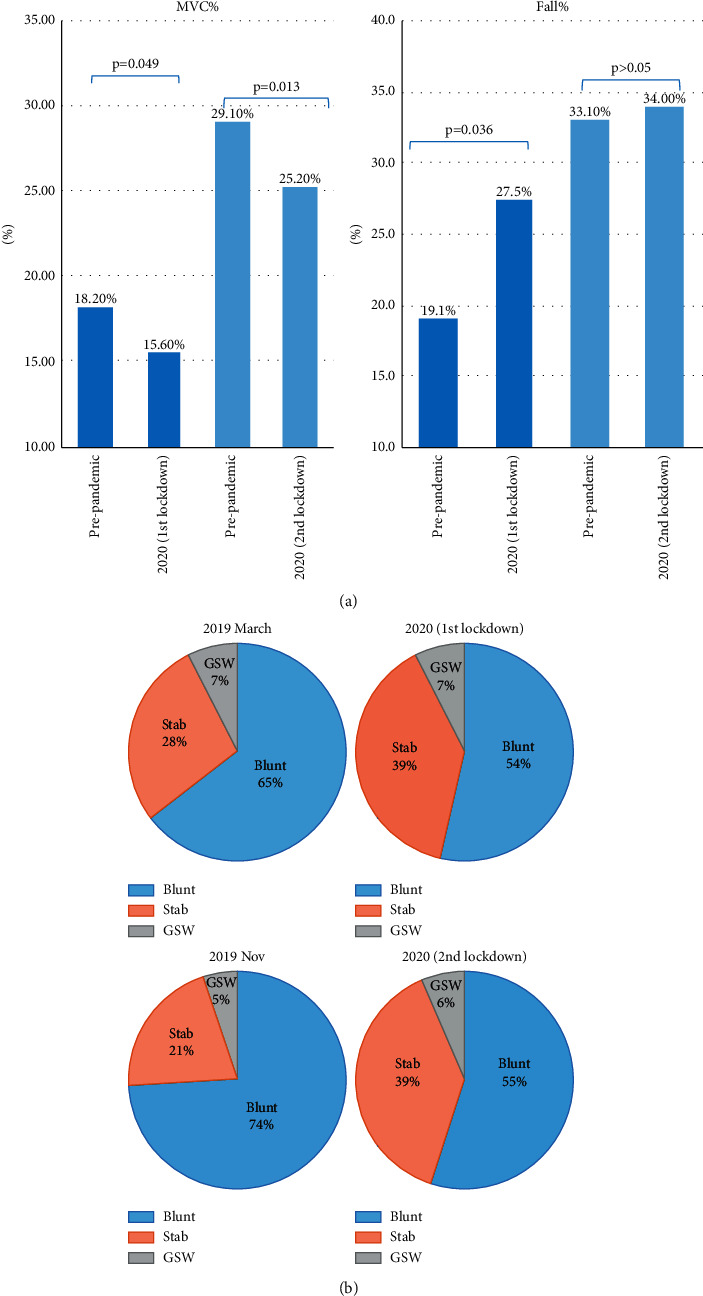
The breakdown of trauma types comparison in our level 1 trauma center in two COVID-19 lockdown periods in 2020 versus prepandemic periods in 2019. (a) The percentage of MVC trauma cases and falls. (b) The percentage of stabbing, blunt trauma, and GSW cases. Multiple comparisons were made using *χ*2 test (chi-square analysis). *p* value refers to a relative portion of MVC/Fall cases to total CT cases.

Trauma cases related to MVCs decreased from 18.2% prepandemic to 15.6% during the 1^st^ lockdown period (*p* = 0.049), while falls increased from 19.1% to 27.5% (*p* = 0.036) (Figure 3(a)) (Table 2). Among crime-related injury, the percentage of stabbing increased from 28% to 39% during the 1^st^ lockdown period and increased from 21% to 39% during 2^nd^ lockdown, while blunt trauma decreased from 65% to 54% and 75% to 65% during two lockdowns (*p* = 0.015) (Figure 3(b)). GSW-related injuries remained similar compared to the prepandemic control months.

## 4. Discussion

This paper provides an analysis of changing patterns in trauma CT imaging during two separate “lockdown” periods associated with the COVID-19 pandemic. Our findings show no significant change in overall trauma initiated CT scans at our institution during the two COVID-19 lockdown periods compared to the prepandemic rate. This is contrary to several other studies which have shown an overall decrease in trauma during pandemic lockdown conditions [9]. One contributing factor could be the type of trauma and patient demographics in our trauma center as compared to other trauma 1 centers. Second, variable regulations during lockdown periods in different regions of Canada and around the world could also contribute to this difference. In addition, the lack of adherence to lockdown principals is possibly another factor that causes major traumatic presentation during lockdown. Although there was no significant change in trauma related CT volumes, we did show a shift in mechanism of injury with decreases in MVC related injuries and increases in other categories, such as falls.

The proportion of trauma-initiated CT scans increased during the initial lockdown period, likely secondary to a decrease in the overall amount of nontrauma-ED-initiated CT scans. We hypothesize that during this period patient without severe illness were reluctant to present to the hospital for fear of contracting the virus, resulting in fewer CT scans being performed overall. This is consistent with the literature [10–12].

The percentage of positive scans increased during the COVID-19 lockdown months. Patients with minor/trivial trauma may have avoided the ED due to fear of contracting the virus while at the hospital [13]. As such, it is actually possible that we have underestimated the true rate of trauma as we only captured trauma patients who received a CT scan. Other studies have shown similar results with a higher proportion of presenting injuries requiring acute care with more severe injuries [13, 14].

A significant increase in falls occurred during both lockdown periods. A variety of factors may have contributed to this increase. Falls commonly occur in the elderly at home, and would likely not be affected by restriction in activities which may have led to a relative increase [15, 16]. Do-it-yourself home renovations have increased during the pandemic which could predispose to trauma, including falls from roofs or latter and/or other unknown mechanisms [17]. The increased rate of falls may have also been secondary to medical decline at home from hospital avoidance and resultant falls at home. Increases in alcohol usage during the pandemic have been demonstrated [18], which may have contributed to increased falls [19, 20]. Syncope has been demonstrated as a presenting feature of SARS-CoV-2 viral infection and may have also contributed to an increased proportion of falls [21, 22].

The rate of CT scans performed for MVCs decreased during the first lockdown of the COVID-19 period. During this time, personal transportation declined significantly due to the promotion of stay-at-home messages and widespread closures during the pandemic [13, 16, 17]. On the other hand, the surge of MVC cases during the 2^nd^ pandemic period was not expected. However, several other factors require consideration. For example, the winter road conditions and decreased daylight hours during this time period are known to cause a significant number of accidents and collision claims in Manitoba and may account for the proportionate increase [6]. Additionally, a recent study by Inada et al. also argued that the lockdown during the COVID-19 pandemic provides an opportunity to speed on empty streets, which may also be a factor in our situation [23].

The percentage of stab injuries significantly increased, while gunshot-related injuries remained the same, and blunt trauma was slightly reduced. No clear reason is identified to explain this phenomenon, and this is possibly an isolated finding specific to our province. Lara-Reyna et al. suggested overall violence-related traumas had increased in relative frequency during the lockdown in New York City [15]. A recent study from Washington has also demonstrated an increase in stabbings and firearm injuries [24]. Our increased rates of stabbing may therefore be a product of the isolation measures, but ultimately, further work will be required to better understand this phenomenon.

### 4.1. Limitations

Our study has several limitations that we acknowledge, including those inherent to a retrospective design. Our data was limited to samples from 1 month portions of each lockdown period. We did not analyze data from time periods during the pandemic with lockdown restrictions partially lifted. Seasonal variability (winter road conditions, ice, and snow) is a known confounder which was not completely accounted for. We also did not analyze the severity of injuries.

## 5. Conclusion

Despite widespread government-mandated closures and strong encouragement for the public to stay at home, the rate of trauma-initiated CT scans at our provincial trauma center did not significantly decrease compared to prepandemic levels. The proportion of CT scans initiated from trauma increased relative to all CTs ordered, and the rate of positive traumatic findings increased during the lockdown periods. There was an increase in the proportion of stabbings and falls, as well as a decrease in MVCs and blunt trauma. The trends and magnitude of the actual imaging utilization data presented will help inform evidence-based decisions for more accurate volume predictions, policy changes, and institutional preparedness for current and future pandemics.

## Figures and Tables

**Table 1:** The comparison of total CTs, total STAT CTs, total trauma patients, total trauma CTs, the rate of pan-scans, and the percentage of negative CT scans in our level 1 trauma center during two lockdown periods in 2020 versus the corresponding prepandemic period in 2019.

	Prepandemic (Mar, 2019)	2020 (1st lockdown)	Prepandemic (Nov, 2019)	2020 (2nd lockdown)	*p* value
Total CTs	3105	2243	3597	3083	0.0008
Total STAT CTs	1832	1573	2303	1973	
STAT CTs rate	59.0%	70.1%	64.0%	64.0%	

Total trauma patients	220	211	281	250	0.0395
Total trauma CTs	436	446	541	508	
Total trauma CT rate	23.8%	28.4%	23.5%	25.7%	

Neg scan	101	84	148	93	0.268
Neg rate	45.90%	39.80%	52.67%	37.20%	

Pan-scan	70	81	107	86	0.832
Pan-scan rate	31.80%	38.40%	38.08%	34.40%	

**Table 2:** The odds ratio with 95% CI of risk factors in first lockdown COVID-19 pandemic.

Risk factor to exposure: COVID-19	OR value (95% CI)	*p* value
Elective vs STAT	0.6138(0.5470–0.6878)	<0.0001
Pan-CT	1.3351(0.8978–1.9853)	0.1534
Negative CT	0.7793(0.5316–1.1423)	0.2013
MV C	0.5571(0.3119–0.9949)	0.049
Fall	1.6066(1.0222–2.52 46)	0.036
Blunt trauma	0.7028(0.4787–1.0317)	0.0718
Stab	1.8936(1.0945–3.2755)	0.0224

## Data Availability

The excel data with deidentification used to support the findings of this study are available from the corresponding author upon request. The original PACS data with patient identification are restricted by the University of Manitoba/Manitoba health policy in order to protect patient privacy. Data are available from the University of Manitoba for researchers who meet the criteria for access to confidential data.
